# *Announcement*: World Day of Remembrance for Road Traffic Victims — November 19, 2017

**DOI:** 10.15585/mmwr.mm6645a5

**Published:** 2017-11-17

In October 2005, the United Nations General Assembly adopted a resolution to mark the third Sunday in November each year as World Day of Remembrance for Road Traffic Victims to honor persons killed or injured in road crashes, recognize their families and communities, and pay tribute to the emergency crews, police, and medical professionals who deal with the traumatic aftermath of road deaths and injuries.*

Road traffic injuries are the ninth leading cause of death worldwide and the leading cause of death among persons aged 15–29 years (*1*). Approximately 1.25 million persons die each year on the world’s roads, and 20 million to 50 million sustain nonfatal injuries (*1*). Although 90% of road traffic deaths occur in low-income and middle-income countries (*1*), approximately 100 persons die and thousands more are injured in motor vehicle crashes every day in the United States (*2*).

A 2016 CDC study found that, among 19 high-income countries, the United States had the most motor vehicle crash deaths per 100,000 persons and per 10,000 registered vehicles, the second highest percentage of deaths involving alcohol-impaired driving, and the third lowest use of front seat belts (*3*). Recent trends do not show evidence of improvement. In 2016, a total of 37,461 persons were killed in road traffic crashes in the United States, a 5.6% increase from 2015 (*2*).

The U.S. Department of Transportation and the National Safety Council have joined with partners, including CDC, to launch the Road to Zero Coalition, with the goal of eliminating U.S. traffic deaths by 2050 (*4*). In the United States alone, implementing proven effective strategies to prevent road traffic deaths can save thousands of lives and hundreds of millions of dollars (*3*). A new Road Safety Annual Report released by the Organisation for Economic Co-operation and Development provides a global perspective. It outlines specific recommendations, potential numbers of lives saved, and potential economic losses prevented in International Road Traffic and Accident Database member and observer countries (*5*).

CDC supports United Nations and World Health Organization measures to dedicate 2011–2020 as the Decade of Action for Road Safety. The program was launched in May 2011 in approximately 100 countries, with the goal of preventing 5 million road traffic deaths globally by 2020. The United Nations is committed to measures to halve the number of global road traffic deaths and injuries by 2020 as one of its Sustainable Development Goals (http://www.un.org/sustainabledevelopment/sustainable-development-goals/). Strategies to support victims and survivors and a guide for nongovernmental organizations are available at http://www.who.int/violence_injury_prevention/publications/road_traffic/ngo_guide/en/.

## Footnotes

* https://www.worlddayofremembrance.org.
